# Quality of life outcomes in colorectal cancer survivors: insights from an observational study at a tertiary cancer center

**DOI:** 10.1007/s11136-025-03918-x

**Published:** 2025-02-18

**Authors:** Pola Marchewczyk, Beatriz Costeira, Francisca Brito da Silva, Daniela Cavadas, Nuno Abecasis, Manuel Limbert, João Maciel

**Affiliations:** 1 Faculdade de Medicina da Universidade de Lisboa, Av. Prof. Egas Moniz, Lisboa, 1649-028 Portugal; 2 Department of General Surgery, Instituto Português de Oncologia de Lisboa Francisco Gentil, R. Prof. Lima Basto, Lisboa, 1099-023 Portugal

**Keywords:** Colorectal cancer, Quality of life, Long-term survivors, Stoma, Rectal cancer, Colon cancer

## Abstract

**Purpose:**

Colorectal cancer (CRC) significantly impacts the quality of life (QoL) of survivors, yet detailed assessments of long-term QoL are sparse. This study evaluates QoL among CRC survivors, examining the influence of different treatments and patient characteristics on outcomes.

**Methods:**

We conducted a cross-sectional study at a tertiary cancer center in Portugal, enrolling CRC patients who underwent curative surgery from 2013 to 2022. QoL was assessed using the EORTC QLQ-C30 and QLQ-CR29 at 1-, 3-, 5-, and 10-year follow-up intervals. Subgroup analyses were performed based on tumor location, radiotherapy administration, chemotherapy administration, presence of a stoma, and time since treatment, with sociodemographic and clinical factors examined on univariate and multivariate analysis.

**Results:**

Of the 825 eligible patients, 324 were invited and 179 participated (response rate: 55.2%). Overall, patients reported high global QoL and functional scores with low symptom scores, comparable to those of the general population. However, rectal cancer survivors experienced poorer outcomes in role and social functioning, body image, and symptom management. Those receiving radiotherapy or chemotherapy reported more symptoms, with chemotherapy recipients showing lower functional scores. Patients with a stoma had significantly lower QoL across functional and symptom scales. Long-term survivors reported decreased physical functioning. Multivariate analysis identified female gender, open surgery, and chemotherapy as factors associated with reduced QoL.

**Conclusion:**

This study highlights significant disparities in QoL outcomes between CRC survivors, with QoL influenced by gender, cancer location, radiotherapy or chemotherapy, stoma presence, and survivorship duration, underscoring the need for personalized support programs and tailored care plans.

**Supplementary Information:**

The online version contains supplementary material available at 10.1007/s11136-025-03918-x.

## Abstract (plain language summary)

People who survive colorectal cancer often face long-lasting health issues that can affect their daily lives. It’s important to understand these challenges so we can find better ways to help them. Our study investigates how different patient characteristics and treatment modalities for colorectal cancer can affect the quality of life afterward. We want to know which of them might make life harder for survivors. The main focus of our research is to see how surgery, radiotherapy, and chemotherapy impact how well survivors can lead their lives, how they feel physically, and their ability to interact socially after cancer. We found that tumor’s location, receiving radiotherapy or chemotherapy, and having a stoma significantly affect life after cancer. For instance, rectal cancer survivors often face harsher quality of life effects than those with colon cancer, particularly in areas like social and role functioning. Furthermore, being a female, undergoing open surgery, and receiving chemotherapy were independently associated to reduced quality of life. These differences underline the importance of considering personal aspects of each patient’s case when planning treatment and support, ensuring that all survivors receive the care and attention they need to improve their overall well-being.

**Supplementary Information:**

The online version contains supplementary material available at 10.1007/s11136-025-03918-x.

## Introduction

Colorectal cancer (CRC) poses significant health challenges globally and remains a predominant cancer type in Portugal, where it is the second leading cause of cancer-related deaths [1]. Advancements in medical science have improved survival rates, however survivors’ long-term quality of life (QoL) has not received comparable attention, despite its paramount importance [2–5]. The impact of CRC and its treatments on QoL is highly variable, influenced by factors such as tumor location, the stage at diagnosis, and the chosen treatment strategies [2–6].

CRC management includes a wide range of treatment modalities such as surgery, chemotherapy, and radiotherapy, each carrying a distinct physical and psychological impact. Especially when applied in combination, they significantly influence patients’ daily functioning and overall well-being [2, 7]. The scope of these effects highlights the need for a thorough assessment of the multidimensional aspects of QoL in CRC survivors [4]. Such evaluations are essential for understanding their recovery trajectories and long-term health outcomes, which could guide the selection and design of targeted interventions, as well as survivorship care plans [7, 8].

Patients with CRC may experience diminished physical functioning and daily life quality due to numerous disease- and treatment-related symptoms, such as pain, change in bowel movements, blood loss and anemia, weight loss, and fatigue. Additionally, their psychological, emotional, social, and role functioning may suffer due to fear, anxiety, sleep disturbances, and depression [6].

Colon cancer patients often report more favorable QoL outcomes compared to those with rectal cancer, who tend to endure more severe symptoms including pelvic pain and complications related to defecation, sexual, and urinary functions [2, 6, 7, 9]. The sphincter-preserving procedures preferred in rectal cancer are associated with their own set of challenges. Approximately 70% of patients undergoing these procedures develop some form of low anterior resection syndrome (LARS), which significantly deteriorates their long-term QoL [10]. Furthermore, although the impact of a stoma on QoL remains a subject of debate in well-designed systematic reviews [11], most recent studies indicate a decline in overall QoL for these patients, particularly in physical and role functioning, and body image [5, 12–14].

Laparoscopic techniques provide several short-term advantages over traditional open surgery, including reduced blood loss, less pain, and shorter recovery times. In the short term, patients undergoing laparoscopic sphincter-preserving procedures report higher QoL scores, including better physical functioning, improved body image, reduced pain, and fewer bowel dysfunction symptoms, compared to those undergoing open surgery [7, 15]. However, a systematic review suggested that differences in QoL may diminish over time as patients who underwent open surgery gradually recover, eventually reaching similar levels of QoL in the long term [15]. Since this review did not include a meta-analysis due to the heterogeneity of the studies analyzed, further investigation is needed to determine whether long-term QoL outcomes truly differ by surgical approach.

Radiotherapy, employed in both neoadjuvant and adjuvant settings for treating rectal cancer, is strongly associated with the development of LARS, as well as urinary and sexual dysfunctions, which significantly reduce QoL [16, 17]. Meanwhile, chemotherapy, generally well tolerated, can lead to adverse effects like neutropenia, diarrhea, skin toxicity, and sensory neuropathy which markedly impair QoL. While most of these side effects resolve after the cessation of treatment, some may persist over the long-term [18, 19].

Remarkably, long-term survivors of CRC often undergo significant psychological adjustments, such as response shift and reframing, that can profoundly influence their perceived QoL, bringing it closer to that of the general population, despite past or ongoing physical and emotional challenges [4–7].

This study aims to assess the QoL of CRC patients treated with curative intent at a tertiary cancer center over a decade. Our secondary objectives include comparing the QoL outcomes to the Portuguese general population, between patients with colon and rectal cancer, patients receiving radiotherapy and those who did not, patients receiving chemotherapy and those who did not, patients with a stoma and those without, and exploring which sociodemographic and clinical variables may influence these outcomes. We intend to improve the understanding of CRC treatment effects, thereby informing future clinical practices and enhancing support mechanisms for patients.

## Methods

### Study design and ethical compliance

A cross-sectional, observational, single-center study was performed to evaluate the QoL of colorectal cancer patients who underwent surgical treatment at our institution from January 2013 to December 2022. Ethical approval was granted by the local ethics committee and all participants provided written informed consent. The study adhered to the STROBE guidelines for reporting observational studies [20].

### Eligibility criteria and patient selection

Adult patients with primary invasive colorectal adenocarcinoma were identified from a prospectively maintained database. Exclusion criteria were R2 resections, multiorgan resection procedures that significantly affect QoL (including total cystectomy and/or sacrectomy), local recurrence, metastatic disease at diagnosis or during follow-up, other malignancies, chronic illnesses severely affecting functionality (e.g., tetraparesis, amyotrophic lateral sclerosis, Parkinson’s disease), and inability to complete the questionnaires due to language barriers, dementia, or death.

Eligible patients were stratified into four cohorts based on the date of surgery, corresponding to follow-up periods of 1-year, 3-year, 5-year, and 10-year, and were invited to participate in the study.

### Quality of life assessment and data verification

QoL was evaluated using the Portuguese validated version of two patient-reported questionnaires: the European Organization for Research and Treatment of Cancer QLQ-C30 (version 3) [21, 22], a QoL instrument that combines both generic and condition-specific measures; and the supplemental EORTC QLQ-CR29 module, specific for colorectal cancer [23]. Scores were processed and missing data managed according to the user manual [24]. QLQ-C30 and QLQ-CR29 scores were normalized to a 0-100 scale, where higher functional scores and lower symptom scores indicate better QoL [21–24]. The EORTC QLQ-C30 summary score was also calculated [24, 25].

During interviews, sociodemographic and clinical data, including gender, age at QoL assessment, employment status, educational level, marital status, tumor location, surgical approach, pTNM stage (AJCC 8th edition), receiving radiotherapy (including those who received radiotherapy alone or as part of a chemoradiotherapy regimen), receiving chemotherapy (including patients who received chemotherapy in either the adjuvant or neoadjuvant setting; while excluding those for whom the chemotherapeutic agent was used solely as radiosensitizer in combination with radiotherapy and did not undergo a proper chemotherapy regimen), and stoma status were confirmed and documented.

### Statistical analysis and outcome measures

Descriptive statistics were used for continuous variables (presented as mean and 95% confidence interval) and frequencies for categorical variables. Subgroup analysis was performed using the Chi-squared test for categorical data and Mann-Whitney U test for continuous variables.

To address potential non-response bias, we performed a comparative analysis of the sociodemographic and clinical characteristics of respondents versus non-respondents.

The mean QoL scores from the EORTC QLQ-C30 for study participants were compared with published means for the Portuguese general population [21]. Differences greater than 10 points were considered clinically meaningful [26, 27]. As the original study data were unavailable and access could not be obtained after contacting the authors, this comparison was restricted to a descriptive visual analysis.

QoL subgroup analyses included comparisons between patients treated for colon and rectal cancer, rectal cancer patients who received radiotherapy versus those who did not, all patients who received chemotherapy versus those who did not, patients with a stoma versus without a stoma, and patients with ‘mid-term’ (1-year and 3-year) versus ‘long-term’ (5-year and 10-year) follow-ups.

To account for potential confounding effects, a multivariable-adjusted logistic regression model was developed to examine associations between QoL and various clinical and sociodemographic factors. The QLQ-C30 summary score [25], categorized into below-median and at/above-median scores, was used as the outcome measure. First, univariate logistic regression was conducted on the entire cohort to identify factors potentially associated with QoL scores. Variables with a p value of less than 0.05 in the univariate analysis were subsequently included in the multivariable logistic regression model to adjust for confounders. Odds ratios (ORs) and 95% confidence intervals (95% CIs) were estimated to identify potential predictors of QoL.

Statistical significance was set at *p* < 0.05 with two-tailed p-values, and all analyses were conducted using IBM^®^ SPSS^®^ Statistics, version 28 [28].

## Results

### Patient recruitment and response rates

**Fig. 1:**
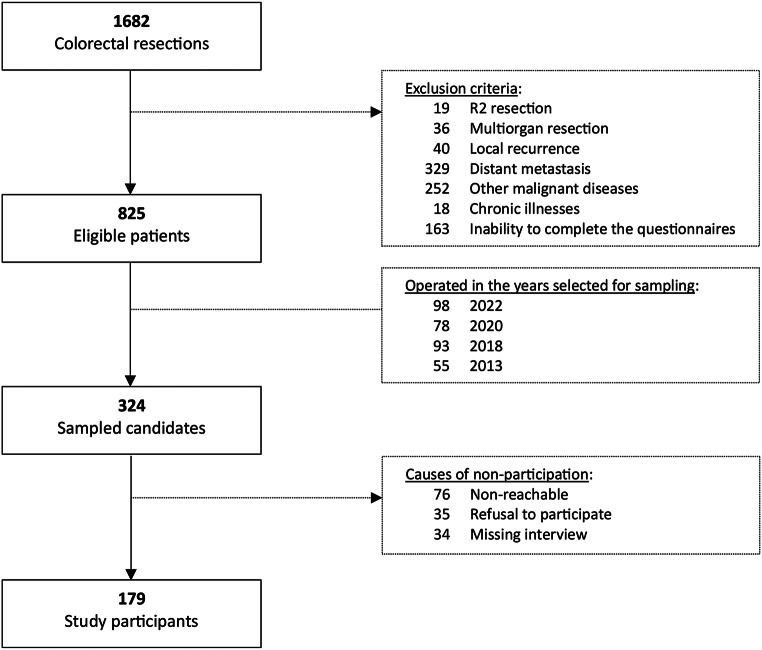
Flow diagram of patients assessed for eligibility and participation in the study

Between 2013 and 2022, a total of 1,682 patients underwent colorectal resection at our institution, with 825 meeting the study’s eligibility criteria. Of these, 324 patients were selected based on follow-up time to assess QoL, with 179 (55.2%) completing the QoL questionnaires, as illustrated in Fig. 1. Participation rates varied by follow-up cohort: 40% at 10-years, 58% at 5-years, 51% at 3-years, and 64% at 1-year.

### Sociodemographic and clinical characteristics

Participants’ sociodemographic and clinical characteristics are provided in Table 1. Comparative analysis of these characteristics between respondents and non-respondents revealed no significant statistical differences (Table S1 of the Supplementary Material).

**Table 1:** Sociodemographic and clinical characteristics of included patients, and comparison between those treated for colon and rectal cancer

Sociodemographic and clinical characteristics, *n* (%)	Total	Colon	Rectal	*p*
	*n* = 179	*n* = 107 (59.8%)	*n* = 72 (40.2%)	
**Gender**				0.544
Male	97 (54.2%)	56 (52.3%)	41 (56.9%)	
Female	82 (45.8%)	51 (47.7%)	31 (43.1%)	
**Age at present (years)**,** median [IQR]**	72 [63–80]	71 [63–78]	68.5 [59.5–74.8]	0.246
**Age at surgery (years)**,** median [IQR]**	66 [58–74]	67 [59–74]	63.5 [58–70]	0.135
**Participation method**				0.189
In person	152 (84.9%)	87 (81.3%)	65 (90.3%)	
Telephone/postal-mail	19 (10.6%)	15 (14%)	4 (5.6%)	
E-mail	8 (4.5%)	5 (4.7%)	3 (4.2%)	
**Employment status**				0.498
Employed	41 (22.9%)	28 (26.2%)	13 (18.1%)	
Unemployed	9 (5.0%)	5 (4.7%)	4 (5.6%)	
Domestic	1 (0.6%)	1 (0.9%)	-	
Retired	128 (71.5%)	73 (68.2%)	55 (76.4%)	
**Educational level**				0.2
Primary school	84 (46.9%)	46 (43%)	38 (52.8%)	
Preparatory school	38 (21.2%)	22 (20.6%)	16 (22.2%)	
High school	29 (16.2%)	20 (18.7%)	9 (12.5%)	
Graduated	22 (12.3%)	13 (12.1%)	9 (12.5%)	
Post-graduated	6 (3.4%)	6 (5.6%)	-	
**Marital status**				0.648
Single	8 (4.5%)	5 (4.7%)	3 (4.2%)	
Married/in a partnership	122 (68.2%)	70 (65.4%)	52 (72.2%)	
Divorced	30 (16.8%)	21 (19.6%)	9 (12.5%)	
Widowed	19 (10.6%)	11 (10.3%)	8 (11.1%)	
**Tumor location**				< 0.001*
Right colon	55 (30.7%)	55 (51.4%)	-	
Left colon	52 (29.1%)	12 (48.6%)	-	
Rectum (upper 1/3)	16 (8.9%)	-	16 (22.2%)	
Rectum (lower 2/3)	56 (31.3%)	-	56 (77.8%)	
**Surgical approach**				0.448
Minimally invasive	121 (67.6%)	70 (65.4%)	51 (70.8%)	
Open	58 (32.4%)	37 (34.6%)	21 (29.2%)	
**pTNM stage**				< 0.001*
I	59 (33%)	24 (22.4%)	35 (48.6%)	
II	68 (38%)	52 (48.6%)	16 (22.2%)	
III	52 (29.1%)	31 (29%)	21 (29.2%)	
**Radiotherapy**				< 0.001*
None	135 (75.4%)	106 (99.1%)	29 (40.3%)	
Radiotherapy	44 (24.6%)	1 (0.9%)	43 (59.7%)	
**Chemotherapy**				0.001*
None	108 (60.3%)	75 (70.1%)	33 (45.8%)	
Chemotherapy	71 (39.7%)	32 (29.9%)	39 (54.2%)	
**Stoma (any time)**				< 0.001*
None	103 (57.5%)	92 (86%)	11 (15.3%)	
Derivative	59 (33.0%)	9 (8.4%)	50 (69.4%)	
Terminal	17 (9.5%)	6 (5.6%)	11 (15.3%)	
**Stoma (at present)**				< 0.001*
None	165 (92.2%)	107 (100%)	58 (80.6%)	
Present	14 (7.8%)	-	14 (19.5%)	

### Quality of life outcomes

In terms of QoL, participants reported high global scores, high functional scores, and low symptom scores, as detailed in Table 2. The highest functional scores observed in the QLQ-C30 were in role and social functioning, with mean scores of 87.1 (95% CI, 83.6–90.5) and 88.4 (95% CI, 85.1–91.6), respectively. The highest functional score in QLQ-CR29 was in body image, with a mean score of 89.6 (95% CI, 87.1–92.2). Anxiety and weight management scored lower, with mean scores of 67.6 (95% CI, 63.2–72) and 75.1 (95% CI, 70.8–79.3), respectively. Sexual interest was low, with mean scores of 38.4 (95% CI, 32.7–44.2) for males and 16.6 (95% CI, 10.8–22.4) for females. As to symptom scores, patients scored the lowest in nausea and vomiting, appetite loss, dyspnea, dysuria, buttock pain, blood and mucus in stool, hair loss, taste, sore skin, stoma care problems, and dyspareunia. Nonetheless, some reported problems like urinary frequency bloating, and impotence each scoring mean scores of 31.8 (95% CI, 28.2–35.5), 25 (95% CI, 20.7–29.2) and 26.4 (95% CI, 20.3–32.6), respectively.

**Table 2:** QoL scores for all participants, and comparison between colon and rectal patients

QoL scores, mean [95% CI]	Total (*n* = 179)		Colon (*n* = 107)		Rectal (*n* = 72)		*p*
**QLQ-C30**							
Global health/QoL	72.3 [69.2–75.3]		74.6 [70.1–78.7]		68.8 [64.2–73.3]		0.033*
*Functional scales*							
Physical functioning	85.4 [82.8–87.9]		86.5 [83.3–89.7]		83.7 [79.5–87.8]		0.185
Role functioning	87.1 [83.6–90.5]		90.5 [86.7–94.3]		81.9 [75.4–88.5]		0.030*
Emotional functioning	80.5 [77.4–83.6]		80.8 [76.7–84.9]		80 [75.2–84.8]		0.622
Social functioning	88.4 [85.1–91.6]		91.4 [87.9–94.9]		83.8 [77.6–90]		0.029*
Cognitive functioning	84.1 [80.9–87.2]		84.4 [80.4–88.4]		83.6 [78.3–88.8]		0.984
*Symptom scales*							
Pain	14 [10.6–17.3]		12 [8.3–15.7]		16.9 [10.5–23.3]		0.659
Fatigue	17.7 [14.5–20.9]		16.9 [13-20.8]		18.8 [13.4–24.3]		0.683
Nausea and vomiting	1.96 [0.7–3.2]		1.7 [0.4-3]		2.3 [0-4.7]		0.911
Appetite loss	5.6 [2.9–8.3]		4.4 [1.4–7.3]		7.4 [2.1–12.7]		0.472
Constipation	12.3 [8.9–15.7]		12.5 [8-16.9]		12 [6.7–17.4]		0.864
Diarrhea	12.9 [9.7–16]		11.8 [7.9–15.8]		14.4 [9-19.7]		0.492
Dyspnea	6.3 [3.8–8.8]		5.9 [2.7–9.2]		6.9 [3-10.9]		0.41
Insomnia	20.1 [16.1–24.1]		22.4 [17-27.8]		16.7 [10.6–22.7]		0.14
Financial difficulties	10.8 [7-14.6]		8.1 [4.2–12]		14.8 [7.4–22.2]		0.155
QLQ-C30 summary score	87.3 [85.5–89.1]		88.1 [85.9–90.4]		86 [83–89]		0.25
**QLQ-CR29**							
*Functional scales*							
Anxiety	67.6 [63.2–72]		69.5 [64–75]		64.8 [57.3–72.4]		0.382
Body image	89.6 [87.1–92.2]		92 [89.3–94.8]		86.1 [81.3–90.9]		0.021*
Weight	75.1 [70.8–79.3]		76 [70.9–81.1]		73.6 [66-81.3]		0.89
Sexual interest							
Male (*n* = 96)	38.4 [32.7–44.2]		35 [27.8–42.2]		33.9 [33.9–52.8]		0.161
Female (*n* = 82)	16.6 [10.8–22.4]		18.6 [10.9–26.3]		13.5 [4.4–22.6]		0.301
*Symptom scales*							
Urinary frequency	31.8 [28.2–35.5]		31 [26.5–35.6]		33.1 [26.8–39.5]		0.76
Urinary incontinence	13.8 [10.2–17.4]		14.3 [9.9–18.8]		13 [6.9–19.1]		0.425
Dysuria	4.5 [2.2–6.7]		4.1 [1.5–6.6]		5.1 [1-9.2]		0.957
Abdominal pain	10.3 [7.4–13.1]		7.8 [4.8–10.8]		13.9 [8.4–19.4]		0.112
Buttock pain	6.7 [4.2–9.2]		3.7 [1.5-6]		11.1 [6-16.2]		0.007*
Bloating	25 [20.7–29.2]		23.7 [18.3–29]		26.7 [19.7–34]		0.551
Blood and mucus in stool	2.9 [1.7–4.1]		1.9 [0.7–3.1]		4.4 [2.1–6.7]		0.030*
Dry mouth	21.2 [17.4–25.1]		19.6 [14.9–24.4]		23.6 [17-30.2]		0.471
Hair loss	4.1 [1.6–6.6]		3.1 [0.6–5.7]		5.6 [0.6–10.5]		0.624
Taste	3.5 [1.6–5.5]		4.7 [2-7.4]		1.9 [0-4.4]		0.107
Flatulence	24 [19.6–28.5]		16.8 [11.8–21.9]		34.7 [27.2–42.2]		< 0.001*
Fecal incontinence	11.9 [8.3–15.6]		3.4 [0.8–6.1]		24.5 [17.1–32]		< 0.001*
Sore skin	8.2 [5.3–11.1]		5.9 [2.9–8.9]		11.6 [5.8–17.3]		0.181
Stool frequency	18.1 [14.9–21.3]		17.1 [13-21.3]		19.4 [14.4–24.5]		0.38
Embarrassment	12.3 [8.7–15.9]		10.9 [6.5–15.4]		14.4 [8.1–20.6]		0.324
Stoma care problems (*n* = 14)	4.8 [0-15.1]		-		4.8 [0-15.1]		-
Impotence (*n* = 97)	26.4 [20.3–32.6]		21 [13.2–28.7]		34.1 [24.2–43.9]		0.017*
Dyspareunia (*n* = 80)	8.4 [3.3–13.6]		6.9 [0.3–13.6]		10.8 [2.2–19.4]		0.289

### Comparison to the Portuguese general population

**Fig. 2:**
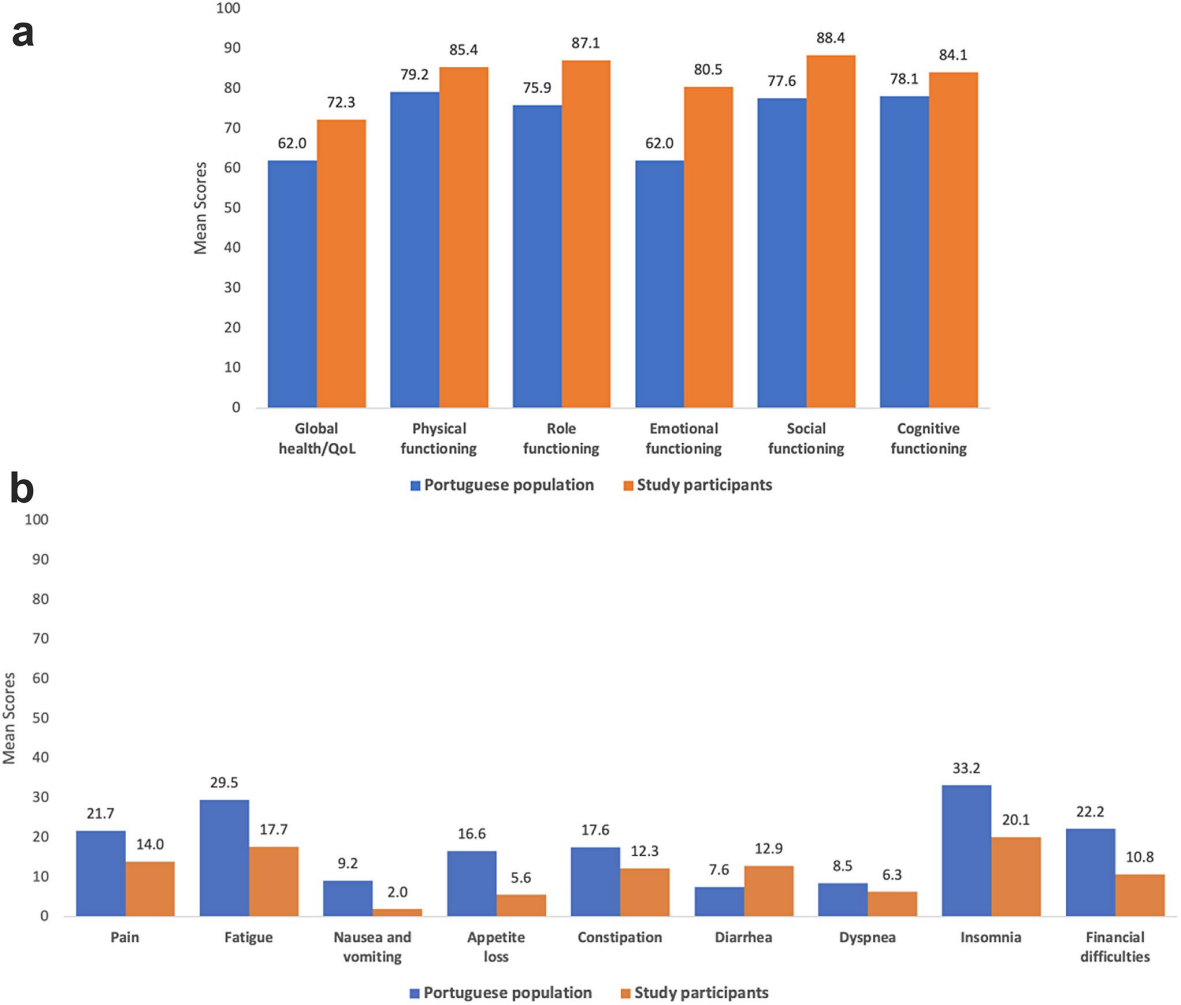
(**a**)Mean global health/QoL and functional scores from the EORTC QLQ-C30 for participants with colorectal cancer compared to mean scores from the general population. (**b**) Mean symptom scores from the EORTC QLQ-C30 for participants with colorectal cancer compared to mean scores from the general population

Compared to the Portuguese general population, study participants generally reported better QoL outcomes, including higher functional and lower symptom scores, as illustrated in Fig. 2a and b. The only exception was diarrhea, where the Portuguese general population achieved more favorable results.

#### Differences in QoL between colon and rectal cancer patients

Significant differences in QoL scores were observed between colon and rectal cancer patients. Rectal cancer patients reported lower global QoL (*p* = 0.033), role functioning (*p* = 0.030), and social functioning (*p* = 0.029), along with poorer scores in body image (*p* = 0.021), buttock pain (*p* = 0.007), blood and mucus in stool (*p* = 0.030), flatulence (*p* < 0.001), fecal incontinence (*p* < 0.001), and impotence (*p* = 0.017).

#### Comparison of QoL in rectal cancer patients between radiotherapy and no radiotherapy

Rectal cancer patients who received radiotherapy reported lower QoL scores on certain symptom scales, specifically for diarrhea (*p* = 0.011), bloating (*p* = 0.034), and impotence (*p* = 0.004), as shown in Table 3. Only 22 rectal cancer patients were treated with surgery alone, without receiving radiotherapy or chemotherapy.

**Table 3:** QoL scores for rectal patients, and comparison between patients who received radiotherapy (RT) and those who did not (no RT)

QoL scores, mean [95% CI]	Rectal (*n* = 72)	RT (*n* = 43)	No RT (*n* = 29)	*p*
**QLQ-C30**				
Global health/QoL	68.8 [64.2–73.3]	69.6 [63.5–75.6]	67.5 [60.3–74.8]	0.609
*Functional scales*				
Physical functioning	83.7 [79.5–87.8]	84 [78.9–89.1]	83.2 [75.8–90.6]	0.77
Role functioning	81.9 [75.4–88.5]	79.8 [71.4–88.3]	85.1 [74.2–96]	0.315
Emotional functioning	80 [75.2–84.8]	78.1 [71.4–84.8]	82.8 [75.9–89.6]	0.375
Social functioning	83.8 [77.6–90]	84.5 [77.1–91.9]	82.8 [71.5–94.1]	0.788
Cognitive functioning	83.6 [78.3–88.8]	84.1 [77.2–91]	82.8 [74-91.5]	0.887
*Symptom scales*				
Pain	16.9 [10.5–23.3]	19 [10.4–27.6]	13.8 [3.8–23.8]	0.392
Fatigue	18.8 [13.4–24.3]	22.5 [14.5–30.4]	13.4 [6.6–20.2]	0.097
Nausea and vomiting	2.3 [0-4.7]	3.8 [0-7.9]	0	0.059
Appetite loss	7.4 [2.1–12.7]	6.2 [0.2–12.2]	9.2 [0-19.3]	0.757
Constipation	12 [6.7–17.4]	12.4 [5.7–19.1]	11.5 [2.4–20.6]	0.727
Diarrhea	14.4 [9-19.7]	20.2 [12-28.3]	5.8 [0.9–10.6]	0.011*
Dyspnea	6.9 [3-10.9]	7.8 [1.9–13.6]	5.8 [0.9–10.6]	0.85
Insomnia	16.7 [10.6–22.7]	20.2 [12-28.3]	11.5 [2.4–20.6]	0.073
Financial difficulties	14.8 [7.4–22.2]	14.7 [5.4–24]	14.9 [2-27.9]	0.826
QLQ-C30 summary score	86 [83–89]	84.6 [80.6–88.5]	88.1 [83.2–93]	0.09
**QLQ-CR29**				
*Functional scales*				
Anxiety	64.8 [57.3–72.4]	60.5 [50.9–70]	71.3 [58.7–83.8]	0.107
Body image	86.1 [81.3–90.9]	86.3 [80.2–92.5]	85.8 [77.8–93.9]	0.782
Weight	73.6 [66-81.3]	68.2 [58-78.5]	81.6 [70.1–93.1]	0.071
Sexual interest				
Male (*n* = 96)	33.9 [33.9–52.8]	47 [34.3–59.7]	39.9 [23.5–54.3]	0.413
Female (*n* = 82)	13.5 [4.4–22.6]	11.1 [0-22.1]	18.2 [0-36.6]	0.407
*Symptom scales*				
Urinary frequency	33.1 [26.8–39.5]	36.1 [27.5–44.6]	28.7 [18.9–38.6]	0.258
Urinary incontinence	13 [6.9–19.1]	14 [5.2–22.7]	11.5 [3–20]	0.954
Dysuria	5.1 [1-9.2]	6.2 [0-12.6]	3.5 [0-7.4]	0.964
Abdominal pain	13.9 [8.4–19.4]	16.3 [8.4–24.2]	10.3 [2.7–18]	0.317
Buttock pain	11.1 [6-16.2]	12.4 [6.1–18.7]	9.2 [0.3–18.1]	0.261
Bloating	26.7 [19.7–34]	32.6 [22.9–42.2]	18.4 [7.9–28.9]	0.034*
Blood and mucus in stool	4.4 [2.1–6.7]	6.2 [2.7–9.8]	1.7 [0-3.7]	0.064
Dry mouth	23.6 [17-30.2]	27.1 [17.6–36.7]	18.4 [9.7–27.1]	0.308
Hair loss	5.6 [0.6–10.5]	6.2 [0–13]	4.6 [0–12]	0.719
Taste	1.9 [0-4.4]	2.3 [0-6.5]	1.2 [0-3.5]	0.825
Flatulence	34.7 [27.2–42.2]	40.3 [29.2–51.4]	26.4 [17.9–35]	0.158
Fecal incontinence	24.5 [17.1–32]	28.7 [18-39.3]	18.4 [8.5–28.3]	0.253
Sore skin	11.6 [5.8–17.3]	12.4 [4-20.8]	10.3 [2.7–18]	0.881
Stool frequency	19.4 [14.4–24.5]	20.2 [13.1–27.2]	18.4 [10.8–26]	0.865
Embarrassment	14.4 [8.1–20.6]	13.2 [4.5–21.9]	16.1 [6.7–25.5]	0.258
Stoma care problems (*n* = 14)	4.8 [0-15.1]	6.7 [0-21.9]	0	0.527
Impotence (*n* = 97)	34.1 [24.2–43.9]	46.3 [32.7–59.9]	18.4 [6.8–30.1]	0.004*
Dyspareunia (*n* = 80)	10.8 [2.2–19.4]	8.4 [0-18.4]	15.2 [0-33.6]	0.418

### Differences in QoL between chemotherapy and no chemotherapy

Patients who received chemotherapy reported lower global health scores, lower functional scores, and higher symptom scores than those who did not, as shown in Table 4. Both the global health score (*p* = 0.024) and the summary score (*p* = 0.004) on the QLQ-C30 were lower among patients who received chemotherapy. Significant differences were also observed in the functional scales of both the QLQ-C30—specifically, role functioning (*p* = 0.008) and emotional functioning (*p* = 0.009)—and the QLQ-CR29, including anxiety (*p* = 0.047) and body image (*p* = 0.007). Additionally, significant differences were found in the symptom scales of both the QLQ-C30 (nausea and vomiting (*p* = 0.047) and financial difficulties (*p* = 0.036)) and the QLQ-CR29 (buttock pain (*p* < 0.001), hair loss (*p* = 0.004), flatulence (*p* = 0.026), and fecal incontinence (*p* = 0.003)).

**Table 4:** QoL scores for all participants, and comparison between patients who received chemotherapy (Chemo) and those who did not (no chemo)

QoL scores, mean [95% CI]	Total (*n* = 179)		Chemo (*n* = 71)		No Chemo (*n* = 108)		*p*
**QLQ-C30**							
Global health/QoL	72.3 [69.2–75.3]		67.7 [62.9–72.6]		75.2 [71.4–79.1]		0.024*
*Functional scales*							
Physical functioning	85.4 [82.8–87.9]		84.7 [80.7–88.7]		85.8 [82.5–89.1]		0.368
Role functioning	87.1 [83.6–90.5]		81.2 [74.8–87.6]		90.9 [87-94.8]		0.008*
Emotional functioning	80.5 [77.4–83.6]		76.1 [71-81.3]		83.3 [79.5–87.1]		0.009*
Social functioning	88.4 [85.1–91.6]		87.3 [82-92.7]		89 [84.9–93.2]		0.35
Cognitive functioning	84.1 [80.9–87.2]		81.5 [76.4–86.5]		85.8 [81.7–89.9]		0.065
*Symptom scales*							
Pain	14 [10.6–17.3]		18.8 [12.4–25.1]		10.8 [7.1–14.5]		0.081
Fatigue	17.7 [14.5–20.9]		20 [14.7–25.4]		16.2 [12.2–20.1]		0.191
Nausea and vomiting	1.96 [0.7–3.2]		3.5 [0.8–6.3]		0.9 [0-1.9]		0.047*
Appetite loss	5.6 [2.9–8.3]		6.1 [1.4–10.8]		5.3 [1.9–8.6]		0.812
Constipation	12.3 [8.9–15.7]		15 [9.6–20.5]		10.5 [6.2–14.8]		0.071
Diarrhea	12.9 [9.7–16]		14.6 [9.6–19.5]		11.8 [7.5–15.9]		0.2
Dyspnea	6.3 [3.8–8.8]		9.9 [4.6–15.1]		4 [1.8–6.3]		0.057
Insomnia	20.1 [16.1–24.1]		20.7 [14.4–30]		19.8 [14.5–25]		0.655
Financial difficulties	10.8 [7-14.6]		14.1 [7.9–20.3]		8.6 [3.9–13.4]		0.036*
QLQ-C30 summary score	87.3 [85.5–89.1]		84.8 [82-87.7]		88.9 [86.5–91.2]		0.004*
**QLQ-CR29**							
*Functional scales*							
Anxiety	67.6 [63.2–72]		62 [54.5–69.4]		71.3 [65.8–76.8]		0.047*
Body image	89.6 [87.1–92.2]		86.1 [81.5–90.7]		92 [89.1–94.9]		0.007*
Weight	75.1 [70.8–79.3]		73.7 [66.5–80.9]		75.9 [70.5–81.3]		0.596
Sexual interest							
Male (*n* = 96)	38.4 [32.7–44.2]		40.9 [31.5–50.4]		36.8 [29.5–44.1]		0.634
Female (*n* = 82)	16.6 [10.8–22.4]		19.8 [8.4–31.2]		14.6 [8.2–21]		0.783
*Symptom scales*							
Urinary frequency	31.8 [28.2–35.5]		31 [24.9–37]		32.4 [27.7–37.2]		0.661
Urinary incontinence	13.8 [10.2–17.4]		14.1 [8.8–19.4]		13.6 [8.7–18.5]		0.392
Dysuria	4.5 [2.2–6.7]		5.6 [0.8–10.4]		3.7 [1.7–5.7]		0.659
Abdominal pain	10.3 [7.4–13.1]		10.8 [5.5–16.1]		9.9 [6.6–13.2]		0.694
Buttock pain	6.7 [4.2–9.2]		12.2 [7-17.4]		3.1 [1-5.1]		<0.001*
Bloating	25 [20.7–29.2]		27.7 [20.4–35]		23.2[17.9–28.4]		0.378
Blood and mucus in stool	2.9 [1.7–4.1]		3.8 [1.7–5.8]		2.3 [0.9–3.7]		0.171
Dry mouth	21.2 [17.4–25.1]		26.3 [19.5–33.1]		17.9 [13.4–22.5]		0.053
Hair loss	4.1 [1.6–6.6]		8 [2.6–13.4]		1.5 [0-3.6]		0.004*
Taste	3.5 [1.6–5.5]		5.2 [1.5–8.9]		2.5 [0.4–4.6]		0.196
Flatulence	24 [19.6–28.5]		30.5 [22.7–38.4]		19.8 [14.6–24.9]		0.026*
Fecal incontinence	11.9 [8.3–15.6]		17.9 [11.1–24.6]		8 [4-12.1]		0.003*
Sore skin	8.2 [5.3–11.1]		10.8 [5.2–16.4]		6.5 [3.3–9.7]		0.327
Stool frequency	18.1 [14.9–21.3]		19 [13.9–24.2]		17.4 [13.3–21.6]		0.543
Embarrassment	12.3 [8.7–15.9]		14.6 [7.4–21.7]		10.8 [6.9–14.7]		0.997
Stoma care problems (*n* = 14)	4.8 [0-15.1]		6.1 [0-19.7]		0		0.602
Impotence (*n* = 97)	26.4 [20.3–32.6]		29.9 [20.3–39.5]		24.1 [15.9–32.2]		0.216
Dyspareunia (*n* = 80)	8.4 [3.3–13.6]		9.7 [0.6–18.7]		7.7 [1.2–14.1]		0.658

### Subgroup analysis based on stoma status

There were 76 patients with a stoma at some point in time; however, currently, only 14 patients have a stoma (13 colostomies and 1 ileostomy), all of whom were treated for rectal cancer. In subgroup analysis, rectal cancer patients with a stoma reported significantly worse outcomes on both functional and symptom scales compared to those without a stoma. Differences were found in role functioning (*p* = 0.034), nausea and vomiting (*p* < 0.001), dyspnea (*p* = 0.047), financial difficulties (*p* = 0.014), anxiety (*p* = 0.030), sexual interest (*p* = 0.038, for females only), urinary incontinence (*p* = 0.041), dysuria (*p* = 0.006), and blood and mucus in stool (*p* = 0.028); details are provided in Table S2 of the Supplementary Material.

### Long-term versus mid-term follow-up comparison

The long-term follow-up subgroup presented significantly lower physical functioning scores (82.8 (95% CI, 78.9–86.7) vs. 87.3 (95% CI, 84-90.5); *p* = 0.029) compared to the mid-term subgroup, as presented in Table S3 of the Supplementary Material. While no significant difference was found in age at the time of surgery between the subgroups (65.5 [IQR 60–72] vs. 66 [IQR 57–74]; *p* = 0.529), the long-term subgroup was significantly older at the time of QoL assessment (72 [IQR 66–79] vs. 68 [IQR 58–76]; *p* = 0.029).

#### Univariate and multivariate analyses

Overall, the median QLQ-C30 summary score for all participants was 91 (IQR 82–96). In univariate analysis, being female (*p* < 0.001), undergoing an open surgical approach (*p* = 0.002), and receiving chemotherapy (*p* = 0.0019) were each significantly associated with lower QoL outcomes. These associations remained significant in the multivariate analysis, with p values of *p* < 0.001, *p* = 0.011, and *p* = 0.034, respectively. Detailed results of both univariate and multivariate logistic regression are presented in Table 5.

**Table 5:** Univariate and multivariate analysis of the sociodemographic and clinical factors

QLQ-C30 summary score	Univariate analysis		Multivariate analysis	
**Sociodemographic characteristics**	OR [95% CI]	p	OR [95% CI]	p
**Gender**				
Male	1 (Ref.)	-	1 (Ref.)	-
Female	0.329 [0.179–0.607]	< 0.001*	0.318 [0.167–0.604]	< 0.001*
**Age at present**	1.006 [0.979–1.032]	0.681	-	-
**Participation method**				
In person	1 (Ref.)	-	-	-
Telephone/postal mail	1.648 [0.616–4.412]	0.32	-	-
E-mail	2.404 [0.452–12.772]	0.303	-	-
**Employment status**				
Employed	1 (Ref.)	-	-	-
Unemployed	0.626 [0.147–2.675]	0.527	-	-
Domestic	0 [0]	1	-	-
Retired	0.887 [0.437-1.800]	0.74	-	-
**Educational level**				
Primary school	1 (Ref.)	-	-	-
Preparatory school	0.826 [0.383–1.780]	0.626	-	-
High school	1.017 [0.435–2.376]	0.969	-	-
Graduated	0.688 [0.268–1.767]	0.438	-	-
Post-graduated	1.652 [0.287–9.516]	0.574	-	-
**Marital status**				
Single	1 (Ref.)	-	-	-
Married/in a partnership	0.756 [0.173–3.303]	0.71	-	-
Divorced	0.459 [0.092–2.280]	0.341	-	-
Widowed	0.540 [0.100–2.930]	0.475	-	-
**Clinical characteristics**				
**Diagnosis**				
Colon	1 (Ref.)	-	-	-
Rectal	0.814 [0.447–1.481]	0.499	-	-
**Tumor location**				
Right colon	1 (Ref.)	-	-	-
Left colon	0.776 [0.362–1.666]	0.516	-	-
Rectum (upper 1/3)	0.559 [0.182–1.720]	0.31	-	-
Rectum (lower 2/3)	0.772 [0.365–1.634]	0.499	-	-
**Surgical approach**				
Minimally invasive	1 (Ref.)	-	1 (Ref.)	-
Open	0.360 [0.188–0.689]	0.002*	0.410 [0.206–0.815]	0.011*
**pTNM stage**				
I	1 (Ref.)	-	-	-
II	1.126 [0.556–2.279]	0.742	-	-
III	0.578 [0.272–1.227]	0.153	-	-
**Radiotherapy**				
None	1 (Ref.)	-	-	-
Radiotherapy	0.590 [0.297–1.172]	0.132	-	-
**Chemotherapy**				
None	1 (Ref.)	-	1 (Ref.)	-
Chemotherapy	0.484 [0.263–0.889]	0.019*	0.491 [0.255–0.949]	0.034*
**Stoma (any time)**				
None	1 (Ref.)	-		-
Derivative	0.966 [0.509–1.834]	0.916	-	-
Terminal	0.982 [0.351–2.745]	0.972	-	-
**Stoma (at present)**				
None	1 (Ref.)	-	-	-
Present	0.463 [0.149–1.441]	0.184	-	-

## Discussion

This study assessed the quality of life (QoL) experienced by mid to long-term survivors of colorectal cancer who received curative treatment at a tertiary cancer center over a decade-long period. We conducted QoL evaluations once for each patient, using a comprehensive set of questionnaires, and strategically selecting individuals at four stages of their follow-up pathway.

Out of the invited cohort, 179 individuals (55.2%) participated in our study. While acknowledging the modest response rate, our figures align with those of similar studies in colorectal cancer [29, 30]. They indicate a consistent pattern of engagement across different follow-up intervals, apart from the ten-year cohort who presented a 40% response rate.

Study participants generally reported favorable QoL outcomes, characterized by good global health, high functional scores, and low symptom scores. Remarkably, these results are comparable to, and in some aspects even better than those of the Portuguese general population, underscoring the resilience and adaptability of cancer survivors. This positive outlook may be influenced by psychological adaptations such as ‘response shift’ and ‘reframing’, where individuals adjust their internal standards of well-being, and ‘rejoice’, a profound sense of gratitude that could significantly enhance QoL perceptions [4, 6, 7].

Distinct variations in QoL were noted between colon and rectal cancer patients. Those with rectal cancer reported lower scores across various domains, including global health, role and social functioning, body image; and specific symptoms such as buttock pain, blood and mucus in stool, flatulence, fecal incontinence, and impotence. These outcomes are consistent with previous research and may be attributed to the aggressive nature of treatments. Irradiation and surgical dissection of the pelvic structures can result in impaired bowel, urinary and sexual functions [2, 6–8].

In a subgroup analysis of rectal cancer patients, those who received radiotherapy reported lower QoL, consistent with previous research [16, 17]. Specifically, radiotherapy was associated with increased complaints of diarrhea, bloating, and impotence.

Patients who received chemotherapy reported lower global health and functional scores, along with higher symptom scores, compared to those who did not receive chemotherapy. These results align with previous research [18, 19], indicating that chemotherapy may negatively impact both overall quality of life and specific aspects such as emotional well-being, body image, and physical symptoms.

The presence of a stoma at the time of QoL assessment was associated with poorer outcomes in our subgroup analysis, affecting both functional and symptom scales. This finding underscores the significant impact of stoma management on patients’ daily lives and overall well-being [12–14].

In a further subgroup analysis, survivors at long-term follow-up intervals (5- and 10-years) reported reduced physical functioning compared to those at mid-term intervals (1- and 3-years). This difference in physical functioning is likely attributable to the older age at which QoL was assessed in the long-term group. Such findings suggest that age-related declines in physical capabilities may have contributed to the diminished QoL reported by these patients.

Our multivariate analysis revealed that women, patients undergoing open surgery, and those receiving chemotherapy reported lower QoL. Previous studies have similarly identified female gender as a predictor of poorer QoL outcomes in colorectal cancer survivors, though the underlying causes are not fully understood [31, 32]. It should be noted that during the period of this study, open surgery was mainly reserved for patients presenting with more extensive locoregional tumor spread, while laparoscopic surgery was preferred in our unit. Additionally, a specific group of patients who underwent chemotherapy reported persistent side effects, especially those associated with neurotoxicity, which may explain the worse QoL outcomes [18–19].

Educational level, employment status, and marital status are commonly used as proxies for socioeconomic status in QoL studies [33–35]. However, in our analysis, none of these factors was significantly associated with the QLQ-C30 summary score. This suggests that socioeconomic status, as measured by these proxies, may not strongly influence QoL outcomes in our cohort. Nevertheless, other socioeconomic factors not assessed in our study, such as income, social support, or access to healthcare, could still play a role and warrant further investigation.

To accurately capture the impact of CRC on survivors, QoL assessments should employ a multi-layered set of instruments that include generic, condition-specific, and disease-specific measures [4, 23, 34]. This comprehensive methodology allowed us to reveal differences not only by tumor location and administration of radiotherapy or chemotherapy but also between patients with and without a stoma, highlighting the value of tailored assessments in uncovering unique patient challenges.

The study is not without limitations. Its cross-sectional design limits our ability to infer causality or track changes in QoL over time. Additionally, the single-center nature of the study may restrict the applicability of our findings to other demographic and clinical settings. A notable concern is the modest response rate; although no statistically significant differences were detected in the characteristics of respondents versus non-respondents, the potential exclusion of more frail individuals– who may have been less willing to participate– could introduce bias into our results. Moreover, the exclusion of patients with metastatic disease at diagnosis or those experiencing recurrence during follow-up may diminish the comprehensiveness of our findings, limiting the integration of QoL assessments with broader oncological outcomes. Finally, the EORTC QLQ-C30 and QLQ-CR29 may not capture all aspects of HRQOL specific to long-term survivors. Future studies could benefit from incorporating the computerized adaptive testing (CAT) version of the EORTC QLQ-C30 [37] once it becomes available in European Portuguese, or the EORTC survivorship core questionnaire [38] once its development is complete, and it has been widely validated for this population.

## Conclusions

In summary, this research highlights the importance of a multi-tiered QoL assessment approach in colorectal cancer survivors, revealing differences that can inform tailored support strategies. The findings advocate for less invasive treatment modalities and the implementation of comprehensive rehabilitation programs to address the QoL challenges identified. Future research should consider a longitudinal, multicentric design that includes a wider spectrum of patients, particularly those with metastatic disease or recurrence, enhancing the applicability and depth of understanding in QoL dynamics among colorectal cancer survivors.

## Electronic supplementary material

Below is the link to the electronic supplementary material.

Supplementary Material 1

## Data Availability

The datasets generated and/or analyzed during the current study are not publicly available due to privacy and ethical restrictions but are available from the corresponding author on reasonable request. Interested researchers may contact the corresponding author to gain access under conditions that adhere to the ethical standards of the research committee and with respect to confidentiality agreements.
