# Supplementary material for: Quality of life outcomes in colorectal cancer survivors: insights from an observational study at a tertiary cancer center
Source: Qual Life Res. 2025 Feb 18;34(5):1501–14. doi: 10.1007/s11136-025-03918-x (PMC12064581; doi:10.1007/s11136-025-03918-x)
Supplement: Supplementary file 1 — Supplementary Material 1 [file 11136_2025_3918_MOESM1_ESM.docx]

**SUPPLEMENTARY MATERIAL**

Table S1 – Sociodemographic and clinical characteristics of the respondents and non-respondents.

| Sociodemographic and clinical characteristics, n (%) | Candidates  n = 324 | Respondents  n = 179 | Non-respondents  n = 145 | | p |
| --- | --- | --- | --- | --- | --- |
| Gender  Male  Female | 177 (54.6%)  147 (45.4%) | 97 (54.2%)  82 (45.8%) | 80 (55.2%)  65 (44.8%) | | 0.860 |
| Age at surgery (years), median [IQR] | 66 [58–74] | 66 [58–73] | 66 [57.5–76] | | 0.692 |
| Age at present (years), median [IQR] | 72 [63–80] | 72 [63–79] | 72 [62–82] | | 0.356 |
| Diagnosis  Colon  Rectal | 199 (61.4%)  125 (38.6%) | 107 (59.8%)  72 (40.2%) | 92 (63.4%)  53 (36.6%) | | 0.500 |
| Tumor location  Right colon  Left colon  Rectum (upper 1/3)  Rectum (lower 2/3)  Missing | 93 (28.7%)  106 (32.7%)  30 (9.3%)  92 (28.4%)  3 (0.9%) | 55 (30.7%)  52 (29.1%)  16 (8.9%)  56 (31.3%)  - | 38 (26.2%)  54 (37.2%)  14 (9.7%)  36 (24.8%)  3 (2.1%) | | 0.333 |
| Surgical approach  Minimally invasive  Open  Missing | 208 (64.2%)  112 (34.6%)  4 (1.2%) | 121 (67.6%)  58 (32.4%)  - | 87 (60%)  54 (37.2%)  4 (2.8%) | | 0.243 |
| pTNM stage  I  II  III  Missing | 104 (32.1%)  113 (34.9%)  103 (31.8%)  4 (1.2%) | 59 (33%)  68 (38%)  52 (29.1%)  - | 45 (31%)  45 (31%)  51 (35.2%)  4 (2.8%) | | 0.351 |
| Radiotherapy  None  Radiotherapy  Missing | 233 (71.9%)  87 (26.9%)  4 (1.2%) | 135 (75.4%)  44 (24.6%)  - | | 98 (67.6%)  43 (29.7%)  4 (2.8%) | 0.238 |
| Chemotherapy  None  Chemotherapy | 185 (57.1%)  139 (42.9%) | 108 (60.3%)  71 (39.7%) | | 77 (53.1%)  68 (46.9%) | 0.191 |
| Stoma (any time)  None  Derivative  Terminal | 190 (58.6%)  107 (33%)  27 (8.3%) | 103 (57.5%)  59 (33.0%)  17 (9.5%) | 89 (61.4%)  46 (31.7%)  10 (6.9%) | | 0.571 |
| Stoma (at present)  None  Present  Missing | 298 (91.9%)  23 (7.1%)  3 (0.9%) | 165 (92.2%)  14 (7.8%)  - | 133 (91.7%)  9 (6.2%)  3 (2.1%) | | 0.745 |

Table S2 – QoL scores for all participants with rectal cancer, and comparison between those with and without a stoma. Men with a stoma: n = 7. Women with a stoma: n = 7.

| QoL scores, mean [95% CI] | Rectal (n = 72) | | No stoma (n = 58) | | Stoma (n = 14) | | p |
| --- | --- | --- | --- | --- | --- | --- | --- |
| QLQ-C30 |  | |  | |  | |  |
| Global health/QoL | 72.3 [69.2–75.3] | | 70.7 [65.7–75.7] | | 60.7 [49.5–72] | | 0.107 |
| Functional scales |  | |  | |  | |  |
| Physical functioning  Role functioning  Emotional functioning  Social functioning  Cognitive functioning | 85.4 [82.8–87.9]  87.1 [83.6–90.5]  80.5 [77.4–83.6]  88.4 [85.1–91.6]  84.1 [80.9–87.2] | | 85 [80.4–89.6]  84.8 [77.7–91.9]  81 [75.9–86.2]  85.3 [80-91.7]  82.5 [76.1–88.8] | | 78.1 [67.5–88.7]  70.2 [53.7–86.8]  75.6 [62.3–88.9]  77.4 [57.9–96.9]  88.1 [80.2–96] | | 0.085  **0.034***  0.262  0.532  0.707 |
| Symptom scales |  | |  | |  | |  |
| Pain  Fatigue  Nausea and vomiting  Appetite loss  Constipation  Diarrhea  Dyspnea  Insomnia  Financial difficulties | 14 [10.6–17.3]  17.7 [14.5–20.9]  1.96 [0.7–3.2]  5.6 [2.9–8.3]  12.3 [8.9–15.7]  12.9 [9.7–16]  6.3 [3.8–8.8]  20.1 [16.1–24.1]  10.8 [7-14.6] | | 15.2 [8.6–21.9]  15.7 [10.6–20.8]  0.3 [0-0.9]  8.6 [2.1–15.1]  13.8 [7.4–20.2]  13.8 [7.9–19.7]  4.6 [1.6–7.7]  17.2 [10.6–23.9]  9.8 [3.9–15.7] | | 23.8 [3.9–43.7]  31.8 [12.8–50.7]  10.7 [0–23]  2.4 [0-7.5]  4.8 [0-11.8]  16.7 [2.1–31.3]  16.7 [0.2–33.1]  14.3 [0-30.7]  35.7 [6-65.4] | | 0.436  0.050  **<0.001***  0.472  0.217  0.732  **0.047***  0.485  **0.014*** |
| QLQ-C30 summary score | 87.3 [85.5–89.1] | | 86.9 [83.7–90.1] | | 82.3 [73.5–91.1] | | 0.210 |
| QLQ-CR29 |  | |  | |  | |  |
| Functional scales |  |  | |  | |  | |
| Anxiety  Body image  Weight  Sexual interest  Male (n = 33)  Female (n = 25) | 64.8 [57.3–72.4]  86.1 [81.3–90.9]  73.6 [66-81.3]  33.9 [33.9–52.8]  13.5 [4.4–22.6] | | 69 [61–77]  88.7 [84.2–93.2]  73.6 [65.4–81.7]  43.5 [33–54]  8 [0.8–15.1] | | 47.6 [28.1–67.2]  75.4 [58.9–91.9]  73.8 [50.9–96.7]  42.7 [13.3–72.1]  33.3 [0-68.9] | | **0.030***  0.058  0.836  0.836  **0.038*** |
| Symptom scales |  | |  | |  | |  |
| Urinary frequency  Urinary incontinence  Dysuria  Abdominal pain  Buttock pain  Bloating  Blood and mucus in stool  Dry mouth  Hair loss  Taste  Flatulence  Fecal incontinence  Sore skin  Stool frequency  Embarrassment  Impotence (n = 33)  Dyspareunia (n = 25) | 33.1 [26.8–39.5]  13 [6.9–19.1]  5.1 [1-9.2]  13.9 [8.4–19.4]  11.1 [6-16.2]  26.7 [19.7–34]  4.4 [2.1–6.7]  23.6 [17-30.2]  5.6 [0.6–10.5]  1.9 [0-4.4]  34.7 [27.2–42.2]  24.5 [17.1–32]  11.6 [5.8–17.3]  19.4 [14.4–24.5]  14.4 [8.1–20.6]  34.1 [24.2–43.9]  10.8 [2.2–19.4] | | 31.9 [25.2–38.6]  10.3 [3.9–16.8]  1.7 [0-3.7]  12.1 [6.7–17.4]  10.9 [5.1–16.7]  29.3 [21.4–37.2]  3.5 [1-5.9]  22.4 [15.6–29.3]  4 [0–9]  0.6 [0-1.7]  36.2 [28-44.5]  23 [15.1–30.9]  9.8 [3.9–15.7]  18.1 [12.6–23.6]  11.5 [5.9–17.1]  32.2 [21.8–42.7]  9.4 [0-18.7] | | 38.1 [18.7–57.5]  23.8 [6.2–41.4]  19.1 [0-38.6]  21.4 [2-40.8]  11.9 [0-24.1]  16.7 [0-34.8]  8.3 [2.1–14.6]  28.6 [7.4–49.7]  11.9 [0-28.1]  7.1 [0-20.6]  28.6 [8.8–48.3]  31 [9-52.9]  19.1 [1-37.1]  25 [11–39]  26.2 [2.1–50.3]  41.5 [9–74]  16.7 [0–46] | | 0.658  **0.041***  **0.006***  0.445  0.756  0.075  **0.028***  0.785  0.055  0.093  0.303  0.557  0.183  0.283  0.349  0.599  0.383 |

Table S3 – QoL scores for all participants, and comparison between mid-term (1- and 3-years) and long-term (5- and 10-years) follow-up patients.

| QoL scores, mean [95% CI] | Total (n = 179) | | Mid-term (n = 103) | | Long-term (n = 76) | | p |
| --- | --- | --- | --- | --- | --- | --- | --- |
| QLQ-C30 |  | |  | |  | |  |
| Global health/QoL | 72.3 [69.2–75.3] | | 72.5 [68.4–76.6] | | 71.9 [67.3–76.5] | | 0.764 |
| Functional scales |  | |  | |  | |  |
| Physical functioning  Role functioning  Emotional functioning  Social functioning  Cognitive functioning | 85.4 [82.8–87.9]  87.1 [83.6–90.5]  80.5 [77.4–83.6]  88.4 [85.1–91.6]  84.1 [80.9–87.2] | | 87.3 [84-90.5]  87.5 [83.1–92]  81.4 [77.7–85.1]  87.2 [82.7–91.8]  86.7 [83.3–90.2] | | 82.8 [78.9–86.7]  86.4 [80.8–92]  79.2 [73.9–84.6]  89.9 [85.3–94.5]  80.5 [74.7–86.2] | | **0.029***  0.776  0.904  0.377  0.169 |
| Symptom scales |  | |  | |  | |  |
| Pain  Fatigue  Nausea and vomiting  Appetite loss  Constipation  Diarrhea  Dyspnea  Insomnia  Financial difficulties | 14 [10.6–17.3]  17.7 [14.5–20.9]  1.96 [0.7–3.2]  5.6 [2.9–8.3]  12.3 [8.9–15.7]  12.9 [9.7–16]  6.3 [3.8–8.8]  20.1 [16.1–24.1]  10.8 [7-14.6] | | 12 [7.7–16.2]  16.9 [12.8–21.1]  2.3 [0.5-4]  4.9 [1.3–8.4]  8.7 [5.3–12.2]  12.6 [8.6–16.6]  5.5 [2.3–8.7]  17.8 [12.7–22.9]  13.3 [7.6–19] | | 16.7 [11.1–22.2]  18.7 [13.8–23.7]  1.5 [0-3.2]  6.6 [2.3–10.9]  17.1 [10.7–23.5]  13.2 [7.9–18.5]  7.5 [3.4–11.5]  23.3 [16.7–29.8]  7.5 [3-11.9] | | 0.212  0.453  0.512  0.349  0.050  0.824  0.302  0.184  0.193 |
| QLQ-C30 summary score | 87.3 [85.5–89.1] | | 88.4 [86.1–90.7] | | 85.7 [82.8–88.7] | | 0.162 |
| QLQ-CR29 |  | |  | |  | |  |
| Functional scales |  |  | |  | |  | |
| Anxiety  Body image  Weight  Sexual interest  Male (n = 96)  Female (n = 82) | 67.6 [63.2–72]  89.6 [87.1–92.2]  75.1 [70.8–79.3]  38.4 [32.7–44.2]  16.6 [10.8–22.4] | | 67.6 [61.8–73.5]  90.1 [87.1–93]  75.7 [70.3–81.1]  39.8 [32.9–46.8]  17.4 [9.4–25.4] | | 67.5 [60.6–74.5]  89 [84.5–93.5]  74.1 [67-81.2]  36.1 [25.8–46.3]  15.8 [7.1–24.5] | | 0.957  0.661  0.997  0.500  0.625 |
| Symptom scales |  | |  | |  | |  |
| Urinary frequency  Urinary incontinence  Dysuria  Abdominal pain  Buttock pain  Bloating  Blood and mucus in stool  Dry mouth  Hair loss  Taste  Flatulence  Fecal incontinence  Sore skin  Stool frequency  Embarrassment  Stoma care problems (n = 13)  Impotence (n = 97)  Dyspareunia (n = 80) | 31.8 [28.2–35.5]  13.8 [10.2–17.4]  4.5 [2.2–6.7]  10.3 [7.4–13.1]  6.7 [4.2–9.2]  25 [20.7–29.2]  2.9 [1.7–4.1]  21.2 [17.4–25.1]  4.1 [1.6–6.6]  3.5 [1.6–5.5]  24 [19.6–28.5]  11.9 [8.3–15.6]  8.2 [5.3–11.1]  18.1 [14.9–21.3]  12.3 [8.7–15.9]  4.8 [0-15.1]  26.4 [20.3–32.6]  8.4 [3.3–13.6] | | 30.1 [25.4–34.8]  11.7 [7.4–15.9]  5.8 [2.5–9.2]  9.1 [5.5–12.6]  6.2 [2.9–9.4]  22 [16.6–27.4]  2.4 [1.3–3.6]  19.7 [15-24.5]  2.6 [0.2-5]  4.9 [2.1–7.7]  23 [17.6–28.4]  10.7 [6.4–15]  7.8 [4.6–11]  17.2 [13.3–21]  12.3 [7.9–16.7]  0 [0]  25.2 [17.4–33]  3.4 [0-7.6] | | 34.2 [28.2–40.2]  16.7 [10.4–23]  2.6 [0-5.4]  11.8 [7.1–16.6]  7.5 [3.6–11.3]  29 [22-35.9]  3.5 [1.2–5.8]  23.3 [16.8–29.7]  6.1 [1.2–11.1]  1.8 [0-4.2]  25.4 [17.9–33]  13.6 [7.1–20.1]  8.8 [3.3–14.3]  19.3 [13.8–24.8]  12.3 [6-18.6]  16.8 [0-70.1]  28.6 [18.1–39.1]  13.4 [4.1–22.7] | | 0.327  0.200  0.075  0.425  0.491  0.117  0.932  0.576  0.362  0.187  0.946  0.821  0.487  0.922  0.293  0.114  0.526  0.096 |
